# Label-Free Detection of Breast Masses Using Multiphoton Microscopy

**DOI:** 10.1371/journal.pone.0065933

**Published:** 2013-06-06

**Authors:** Xiufeng Wu, Gang Chen, Jianping Lu, Weifeng Zhu, Jingting Qiu, Jianxin Chen, Shusen Xie, Shuangmu Zhuo, Jun Yan

**Affiliations:** 1 Department of Surgery, Fujian Provincial Tumor Hospital, Teaching Hospital of Fujian Medical University, Fuzhou, Fujian, People’s Republic of China; 2 Department of Pathology, Fujian Provincial Tumor Hospital, Teaching Hospital of Fujian Medical University, Fuzhou, Fujian, People’s Republic of China; 3 Institute of Laser and Optoelectronics Technology, Fujian Provincial Key Laboratory for Photonics Technology, Key Laboratory of OptoElectronic Science and Technology for Medicine of Ministry of Education, Fujian Normal University, Fuzhou, People’s Republic of China; 4 Fujian University of Traditional Chinese Medicine, Fuzhou, P. R. China; Health Canada, Canada

## Abstract

Histopathology forms the gold standard for the diagnosis of breast cancer. Multiphoton microscopy (MPM) has been proposed to be a potentially powerful adjunct to current histopathological techniques. A label-free imaging based on two- photon excited fluorescence and second-harmonic generation is developed for differentiating normal breast tissues, benign, as well as breast cancer tissues. Human breast biopsies (including human normal breast tissues, benign as well as breast cancer tissues ) that are first imaged (fresh, unfixed, and unstained) with MPM and are then processed for routine H-E histopathology. Our results suggest that the MPM images, obtained from these unprocessed biopsies, can readily distinguish between benign lesions and breast cancers. In the tissues of breast cancers, MPM showed that the tumor cells displayed marked cellular and nuclear pleomorphism. The tumor cells, characterized by irregular size and shape, enlarged nuclei, and increased nuclear-cytoplasmic ratio, infiltrated into disrupted connective tissue, leading to the loss of second-harmonic generation signals. For breast cancer, MPM diagnosis was 100% correct because the tissues of breast cancers did not have second-harmonic generation signals in MPM imaging. On the contrary, in benign breast masses, second-harmonic generation signals could be seen easily in MPM imaging. These observations indicate that MPM could be an important potential tool to provide label-free noninvasive diagnostic impressions that can guide surgeon in biopsy and patient management.

## Introduction

Breast cancer is among the most common cancer in women worldwide and ranks second as a cause of cancer death in women,who are nearly 100 times more likely to develop breast cancer than men [1]. While physical breast exam, mammography, ultrasound, and other breast imaging methods can help detect a breast abnormality, biopsy followed by pathological analysis is the only definitive way to determine if cancer is present. Pathology examination remains the gold standard for diagnosis and surveillance of breast cancer. However, there are several drawbacks. First, the routine pathology examination involving fixation, slicing, staining, and finally being examined under a microscope by pathologists to make a diagnosis, results in a turnaround time ranging from hours to days. This time-consuming technique has the disadvantage of prolonging the surgical procedure and thereby the time the patient stays under general anesthesia, increasing patient morbidity as well as cost of the procedure [2]. In addition, the morphologic artifacts associated with processing procedures used in histology are always of concern. Furthermore, patients with suspicious breast masses or positive margin may be subjected to numerous biopsies or re-excised to rule in or out carcinoma, which results in diagnosis delays or misdiagnosis. Lastly, the breast is not a good model for frozen section analysis because fatty tissue does not perform well in this technique [3]. Therefore, it is imperative that a new rapid and label-free noninvasive diagnostic method for breast cancer detection is much needed to reduce the resulting mortality rate.

The recent invention of multiphoton microscopy has opened up new possibilities for the in vivo assessment of tissue [4]. This high-magnification, high-resolution ‘optical biopsy’ techniques are currently being explored as a label-free noninvasive diagnostic approach. It has many unique advantages including inherent optically sectioning, deep optical penetration, and reduced specimen photobleaching and photodamage compared with confocal laser microscopy [5]. Most importantly, MPM enables detailed visualization of cellular and subcellular structures based on two-photon excited fluorescence (TPF) or second-harmonic generation (SHG), which is important because changes in cellular and subcellular morphology are associated with the development of precancerous lesions and cancers, and has been widely used in the study of biomedicine areas [6]–[11]. Especially, recent studies showed that the miniaturized MPM and multiphoton probe allow the clinical use of multiphoton endoscopy for diagnosing cancer [12]–[20]. Therefore the ability of MPM to evaluate relevant breast lesions, in comparison with the current gold standard (H-E staining) must be assessed. In our study, the assessment of breast masses (including normal breast tissues, fibrocystic breast disease tissues, fibroadenoma tissues, breast cancer tissues) by means of multiphoton laser imaging with measurement of SHG and TPF is performed. We compare the acquired images from cancerous tissues and the normal and benign tissues to determine whether this method can be used to make label-free diagnosis of breast cancer and to assess its potential for the noninvasive in vivo pathophysiological analysis of breast lesions. This is, to our best knowledge, the first demonstration of the use of MPM to label-freely image breast masses.

## Materials and Methods

### Ethics Statement

Patients with breast masses were recruited to participate in this study, which was approved by Institutional Review Board of Fujian Provincial Tumor Hospital. Written informed consent was obtained prior to study participation.

### Sample Preparation

Breast tissues were obtained from female patients undergoing surgical biopsy and surgery at Fujian Provincial Tumor Hospital. The excised tissues were immediately snap-frozen in liquid nitrogen for storage. A total of eighteen patients were enrolled in this study, including 6 cases of fibroadenoma, 6 cases of fibrocystic disease, and 6 cases of breast cancer. Six normal tissue samples were also collected from the same patients with breast cancer. Samples were snap-frozen and kept in liquid nitrogen (−196°C) until use. The tissue sections for multiphoton imaging were cut into pieces approximately 2×1×1 cm in size and placed in the Glass Bottom Dish (MatTek, coverglass: 0.085–0.13 mm). A total of 24 tissue section samples were analyzed in this study. The sample (approximately 2×1×1 cm in size) was imaged with the cover glass facing the microscope objective. Moreover, to avoid dehydration or shrinkage during the imaging process, a little phosphate-buffered saline (PBS) solution was dripped into the tissue specimen. Immediately after MPM imaging (up to a maximum of 1 hour), samples were fixed in 10% buffered formalin and submitted for routine histopathology (including sectioning, staining, assessment under microscope).

### MPM Examination

The MPM system used in this study has been described previously [21]. The MPM system contained a high-throughput scanning inverted Axiovert 200 microscope (Zeiss LSM 510 META, Jena, Germany) and a mode-locked femtosecond Titanium: sapphire (Ti:s) laser (110 fs, 76 MHz), tunable from 700 nm to 980 nm (Coherent Mira 900-F, Coherent Inc., Santa Clara, CA, USA). For high-resolution imaging, a high-numerical-aperture, oil immersion objective (Plan-Apochromat 63×, N.A.1.4, Zeiss) was employed in MPM examination. In the multichannel mode, the backward SHG and two photon-excited fluorescence (TPEF) signals from the tissue sample were collected by the META detector, which had eight independent-channels and each channel could selectively be set to detect emission signals within the random range from 377 nm to 716 nm to achieve imaging. One channel was corresponding to the wavelength range of 387 to 409 nm to show microstructure of tissue component from SHG signals (red color-coded), whereas another channel was covered with the wavelength range from 430 to 708 nm to present the morphology of tissue component from TPEF signals (green color-coded). The excitation wavelength λex used in this study is 800 nm. All the images had a 12-bit pixel depth. The images were obtained at 2.56 µs per pixel.

### Histopathology

After multiphoton imaging, histological procedures were completed including formalin fixation, paraffin embedding, 5-µm-thick sectioning. Paraffin-embedded sections were stained with hematoxylin-eosin (H-E) and examined by light microscopy.

## Results

### MPM Diagnostic Features for Breast Masses

**Figure 1:**
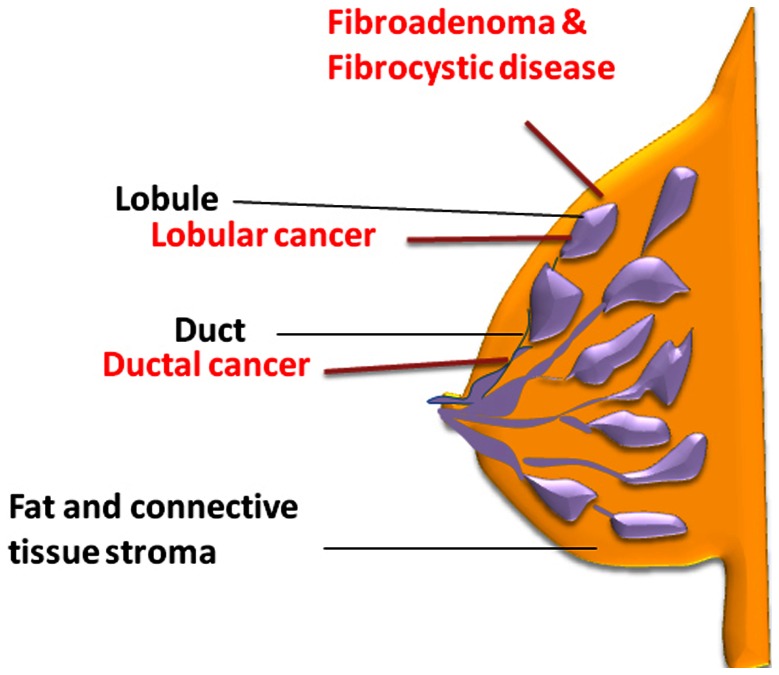
Schematic illustration of normal breast structure. The main parts of the breast are lobules, ducts, and stroma (fatty tissue and connective tissue surrounding the ducts and lobules). Most breast cancers begin in the ducts (ductal), some in the lobules (lobular) and the rest in other tissues. Fibrocystic breast disease and fibroadenoma usually develop within the breast lobules.

**Figure 2:**
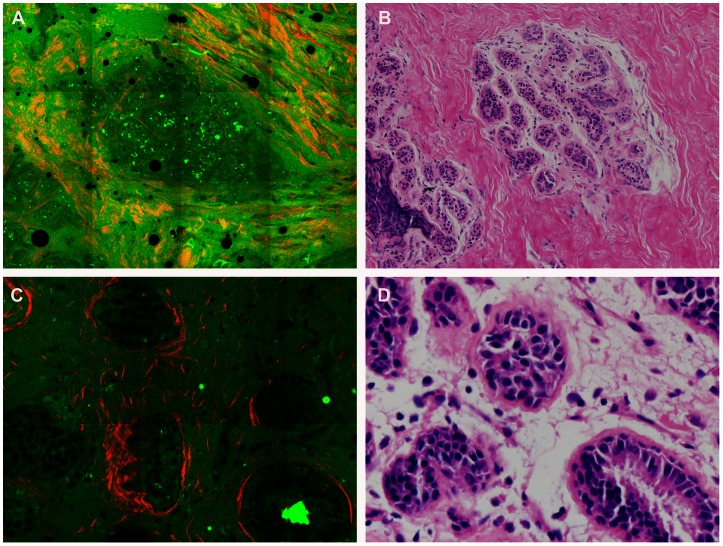
Normal breast tissue. Multiphoton microscopy (MPM) image (A) and corresponding hematoxylin-eosin (H-E) image (B) at low magnification show normal breast is predominantly consist of adipose and fibrous structures surrounding ducts. The MPM image (C) and corresponding H-E image (D) at high magnification show ducts are lined by simple columnar epithelium and basement membrane. In MPM image, the red color represents the SHG signal from collagen, and the green color represents TPEF from autofluorescence in cells. (Original magnifications 63x [A, C]; 20x [B]; 40x [D]).

**Figure 3:**
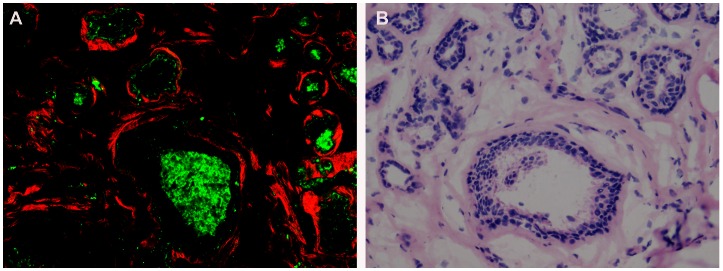
Fibrocystic breast disease. Multiphoton microscopy (MPM) image (A) and corresponding hematoxylin-eosin (H-E) image (B) show fibrocystic breast changes are characterized by an increase in the number and size of glandular tissues. These include benign fibrous tissues and scattered cysts containing amorphous material. (Original magnifications 63x [A]; 20x [B]).

**Figure 4:**
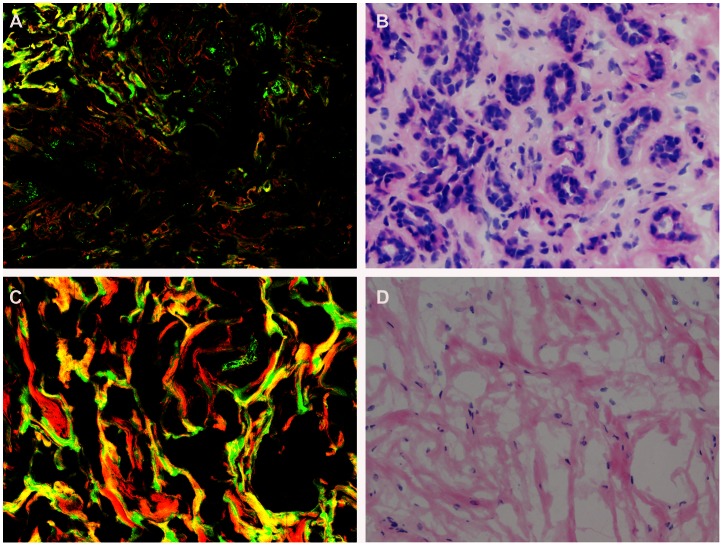
Fibroadenoma. Multiphoton microscopy (MPM) image (A) and corresponding hematoxylin-eosin (H-E) image (B) show the unique morphological structure of fibroadenoma with the acinar arrangement of the round and ovoid tubules, surrounded by a myoepithelial layer and the stromal component. The stroma is made up of loose connective tissue(C, D). (Original magnifications 63x [A, C]; 20x [B, D]).

**Figure 5:**
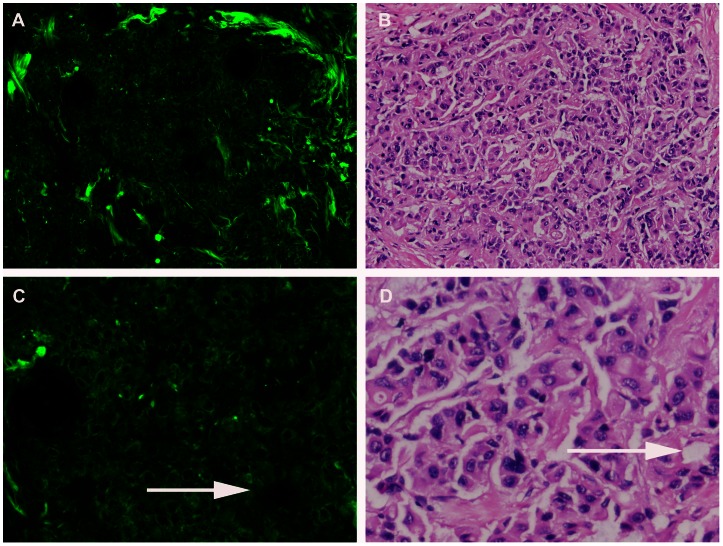
Breast cancer. Multiphoton microscopy (MPM) image (A) and corresponding hematoxylin-eosin (H-E) image (B) show cancer cells displayed marked cellular and nuclear pleomorphism. Cancer cells are characterized by irregular size and shape, enlarged nuclei, and increased nuclear-cytoplasmic ratio. Glandular structure consisting of tumor cells is evident (as indicated by arrow) shown in C, D). (Original magnifications 63x [A]; 20x [B]).

Based on multiphoton images obtained, we were able to differentiate normal breast tissues, benign, as well as breast cancer tissues. Normal breast tissues consisted of the ducts and glands (which maked up the bulk of the volume of the adult breast) as well as adipose and fibrous structures in varying proportions, as shown in Fig. 1. These structures possess strong multiphoton signals and can be clearly recognized in multiphoton images, as shown in Fig. 2A. At high magnification, ducts lined by simple columnar epithelium and obvious basement membrane were identified in the normal tissues with multiphoton microscopy, mainly because of the SHG signals from collagen components, as shown in Fig. 2C. All these features were present in the H-E stained image shown in Fig. 2B, D. Fibrocystic breast changes were characterized by an increase in the number and size of glandular tissues, usually within the breast lobules. These included benign fibrous tissues and scattered cysts containing amorphous material. The cyst lining was flattened or absent in some cases. These features were distinctly observed in the multiphoton image and H-E stained image shown in Fig. 3A, B respectively. It was seen in Figure 4A that fibroadenoma showed unique morphological structure with the acinar arrangement of the round and ovoid tubules, surrounded by a myoepithelial layer and the stromal component. The stroma is made up of loose connective tissue (as shown in Fig. 4C). The same pattern was also clearly identified in the H-E stained image, as shown in Fig. 4B and 4D. Figure.5A showed multiphoton image of breast cancer. The tumor cells displayed marked cellular and nuclear pleomorphism. The tumor cells, characterized by irregular size and shape, enlarged nuclei, and increased nuclear-cytoplasmic ratio, infiltrated into disrupted connective tissue, leading to the loss of basement membrane (no SHG signals). Glandular structure consisting of tumor cells was evident, as indicated by the arrow in the multiphoton image shown in Fig. 5C. These features were consistent with that in the H-E stained image shown as Fig. 5D. Comparative features between H-E and MPM were shown in Table 1.

**Table 1:** Comparative Features Between Hematoxylin-Eosin (H-E) and MPM.

Breast Lesions	Diagnostic Features Identifiable on Both H-E and MPM	MPM Limitations
Fibrocystic breast changes	1. Increase in the number and size of glandular tissues.2. Benign fibrous tissues and scattered cysts containing amorphous material.	Absence of intranuclear details
Fibroadenoma	1. Acinar arrangement of the round and ovoid tubules, surrounded by amyoepithelial layer and the stromal component.2. The stroma is made up of loose connective tissue.	Absence of intranuclear details
Breast cancer	1. Marked cellular and nuclear pleomorphism.2. Cancer cells with irregular size and shape, enlarged nuclei, and increasednuclear-cytoplasmic ratio.3. Glandular structure consisting of tumor cells.	Failure to assess presence of muscularis, including involvement by tumor, due to penetration-depth limitations in current MPM imaging instrumentation.

### MPM Diagnostic Accuracy for Breast Masses

In this study, 24 samples were collected, which included 6 cases of fibroadenoma, 6 cases of fibrocystic disease, 6 cases of breast cancer, and 6 cases of normal tissues. MPM diagnosis was compared with golden standard H-E diagnosis. The sensitivity, specificity, accuracy, positive predictive value, and negative predictive value of MPM diagnosis was 88.89%, 83.33%, 87.50%, 94.12%, and 71.43%, respectively. The discrepancy in MPM and H-E comparison was mainly shown in normal tissue and fibrocystic disease. False negative rate was 11.11% (2/18) because 2 cases of fibrocystic disease were diagnosed as normal tissue, while false positive rate was 16.67% (1/6) because one sample of normal tissue was diagnosed as fibrocystic disease. For breast cancer, MPM diagnosis was 100% correct because the tissues of breast cancers did not have second-harmonic generation signals in MPM imaging. On the contrary, in benign breast masses, second-harmonic generation signals could be seen easily in MPM imaging. The details of MPM diagnostic accuracy were shown in Table 2.

**Table 2:** MPM diagnostic accuracy.

N = 24			H-E diagnosis				
			Abnormal (N1 = 18)			Normal (N2 = 6)	
			Fibroadenoma (n = 6)	Fibrocystic disease (n = 6)	Breast cancer (n = 6)		
MPM diagnosis	Abnormal (N3 = 17)	Fibroadenoma (n = 6)	6	0	0	0	PPV = 16/17 = 94.12%
		Fibrocystic disease (n = 5)	0	4	0	1	
		Breast cancer (n = 6)	0	0	6	0	
	Normal (N4 = 7)		0	2	0	5	NPV = 5/7 = 71.43%
			Sens = 16/18 = 88.89%			Spec = 5/6 = 83.33%	Accuracy = (16+5)/24 = 87.50%

Abbreviations:

MPM: Multiphoton microscopy;

H-E: Hematoxylin-eosin;

Sens: Sensitivity;

Spec: specificity;

PPV: Positive predictive value;

NPV: Negative predictive value.

## Discussion

In this study, we have demonstrated the feasibility of using MPM to distinguish breast cancers from normal tissues and benign lesions. High-contrast, high-resolution images are obtained from normal, benign (fibroadenoma, fibrocystic disease), breast cancer tissues by using MPM based on TPEF and SHG. Our results show that MPM has the ability to characterize breast tissue structures and cell morphology in a manner similar to traditional histological analysis. On MPM images, normal breast tissues present predominantly adipose and fibrous structures surrounding ducts. By comparison, fibrocystic disease possesses unique morphological features on MPM images including benign fibrous tissues and scattered cysts containing amorphous material which are in accordance with pathological criteria. Furthermore, on MPM images, fibroadenoma shows the morphology with acinar arrangement of the round and ovoid tubules, surrounded by a myoepithelial layer and the stromal component. The stroma is made up of loose connective tissue. On the other hand, cancer tissues exhibit distinct cellular features with high cellularity. Most importantly, at high magnification, the pleomorphism of the carcinoma cells, as well as the neoplastic cells infiltrating through the stroma, could be seen with breast cancer. Glandular structure consisting of tumor cells is another feature in breast cancer. These disease-related features can be used to distinguish cancer lesions from normal and benign tissues.

Our results show a good distinction of breast cancer from normal tissues and benign lesions. Compared to H-E analysis, however, our approach presents a much faster strategy without the addition of exogenous fluorophores and damaging histological procedures, thus significantly resulting in reduced diagnostic time and increased accuracy. Currently, histopathology is the gold standard for diagnosing breast cancer. However, the routine histopathological procedure is time-consuming, which includes 10% buffered formalin processing, paraffin embedding, sectioning at 5 mm, and then hematoxylin-eosin (H-E) staining. This procedure usually needs 3–5 days, and sometimes the result is ambiguous or negative because of too little sample or tissue necrosis. Therefore, the availability of a noninvasive diagnostic tool that can obtain histopathological analysis comparable to H–E stained histopathology would be of extraordinary benefit to the medical community. Our results showed that multiphoton imaging provided the ability to detect cellular and subcellular details in fresh, unfixed, unstained breast specimens, with image quality that was comparable to H–E stained imaging. This new technology is coming up and we might see this real-time diagnosis in the near future.

In addition, label free imaging of tissue with subcellular resolution yields important clinical implications, including (i) rapidly non-invasive in vivo detection of breast cancers based on cellular characterization. Breast is particularly accessible to optical techniques. MPM apparatus miniaturization or integration of MPM into the intra-fiberoptic ductoscopy or transdermal biopsy needle would have the advantage of providing immediate noninvasive in vivo diagnosis, which avoids repeated needle or surgical biopsy and reduces patient anxiety in awaiting results and expedites the scheduling of procedures in cases of carcinoma. (ii) intraoperative real time margin assessment in the operating room, avoiding the need for reexcision surgeries resulting from positive margins. For example, negative margin is critical for a low rate of cancer recurrence during breast-conserving procedures, where clusters of tumor cells can be easily identified in adipocyte-rich milieu [22]. (iii) direct in situ assessment of the therapeutic effectiveness at a cellular level.

In this study, the MPM images alone provided sufficient detail to classify most lesions as either benign or neoplastic using the same basic diagnostic criteria as histopathology (architecture and cell morphology). Different features have not yet been observed so far. However, MPM imaging has the potential to provide real-time diagnosis for breast masses. Moreover, MPM imaging is very sensitive to collagen tissue. Therefore, in normal breast tissue, MPM imaging can show the basement membrane. On the contrary, in breast cancer which cancer cells disrupt, or replace the collagen, resulting in the loss of basement membrane, MPM imaging can not show the basement membrane. This is the advantage of MPM imaging over traditional H-E staining. Currently, this is a pilot study which investigated the feasibility of using MPM to diagnose breast masses. In this pilot study, a total of eighteen patients were enrolled, including 6 cases of fibroadenoma, 6 cases of fibrocystic disease, 6 cases of breast cancer. Six normal tissue samples were also collected from the same patients with breast cancer. The purpose of this pilot study was to compare the acquired images from cancerous tissues and benign tissues to determine whether this method can be used to make label-free diagnosis of breast cancer and to assess its potential for the noninvasive in vivo pathophysiological analysis of breast lesions. Our results showed that MPM could characterize breast tissue structures and cell morphology in a manner similar to traditional histological analysis. In the tissues of breast cancers, MPM showed that the tumor cells displayed marked cellular and nuclear pleomorphism. The tumor cells, characterized by irregular size and shape, enlarged nuclei, and increased nuclear-cytoplasmic ratio, infiltrated into disrupted connective tissue, leading to the loss of collagen, which means no second-harmonic generation signals. For breast cancer, MPM diagnosis was 100% correct because the tissues of breast cancers did not have second-harmonic generation signals in MPM imaging. On the contrary, in benign breast masses, second-harmonic generation signals could be seen easily in MPM imaging. Overall, we believe this modality has a promising future to facilitate diagnosis of breast cancer.

The use of MPM does have some limitations in its current state. For instance, the penetration depth of MPM imaging will be important challenges to address in this development, which limits evaluation of deeper tissue change. Furthermore, utilization of high NA objectives in MPM systems results in smaller fields of view, which may contribute to missing diseased tissues. Lastly, expensive systems currently limit its widespread application in clinical setting.

In short, the establishment of diagnostic features is essential and significant for developing multiphoton endoscopy to facilitate diagnosis of breast masses. In this pilot study, we established diagnostic features for identifying the normal breast tissues, benign, as well as breast cancer tissues by investigating their multiphoton microscopic images. In the future work, a large-scale study will be conducted to determine sensitivity, specificity, negative predictive value, positive predictive value with respect to traditional histological analysis. This pilot study has established the diagnostic features for breast masses, which is very helpful for further blinded test for diagnostic accuracy.

### Conclusions

We demonstrated that, for the first time, the feasibility of MPM based on TPEF and SHG to differentiate breast cancer from normal and benign lesions. The unique capability of MPM to perform contrast and optical sectioning imaging with high resolution opens new possibilities for in vivo and real-time diagnosis of breast cancer without the need for histological staining or administration of exogenous contrast agents. In particular, the ability to visualize basement membrane with high resolution with MPM is a key advance in the field that allows dynamic monitoring of preinvasive disease during cancer progression as the breast cancer invasion and metastasis are associated with decomposition of basement membrane (mainly consisting of collagen) [23], [24]. The resulting structural changes in the collagen and possible decomposition of collagen lead to changes in stromal scattering [25]–[29]. Thus, the characterization of intrinsic SHG signals from basement membrane may be of interest for evaluation of potential metastasis in breast cancer. In the future work, we would focus on evaluation of the morphologic changes of basement membrane in different breast cancer stages.

In concert, although pathologic examination would remain the gold standard of care for breast cancer surgery, we have good reason to believe that MPM has the potential to improve in vivo real- time noninvasive diagnosis of breast masses.
